# Supplementary figures and images for: High-resolution, genotype-free mapping of genetic variation with CRI-SPA-Map
Source: bioRxiv. 2025 Sep 18:2025.09.16.676640. Preprint. [Version 1] doi: 10.1101/2025.09.16.676640 (PMC12458176; doi:10.1101/2025.09.16.676640)

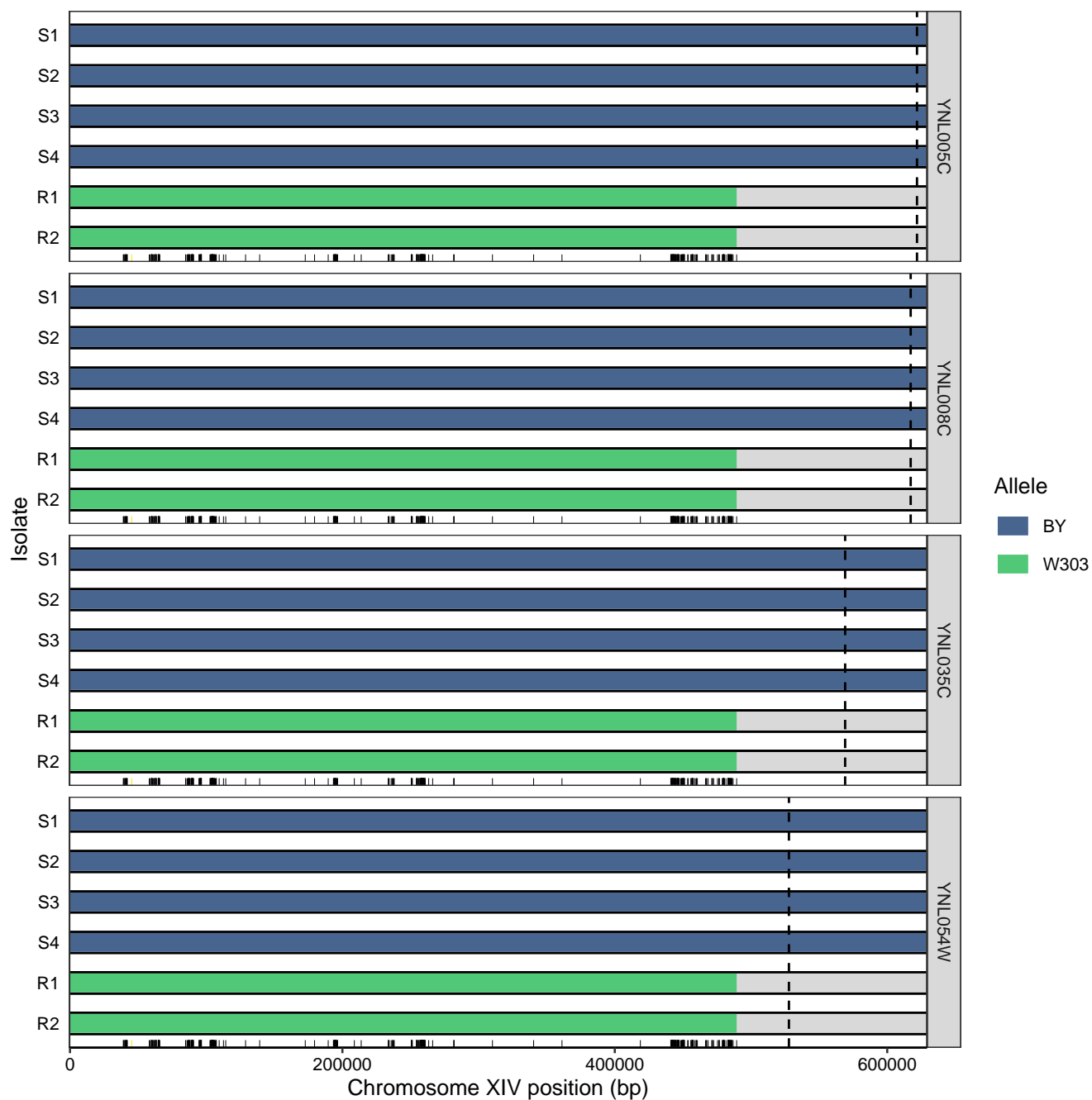

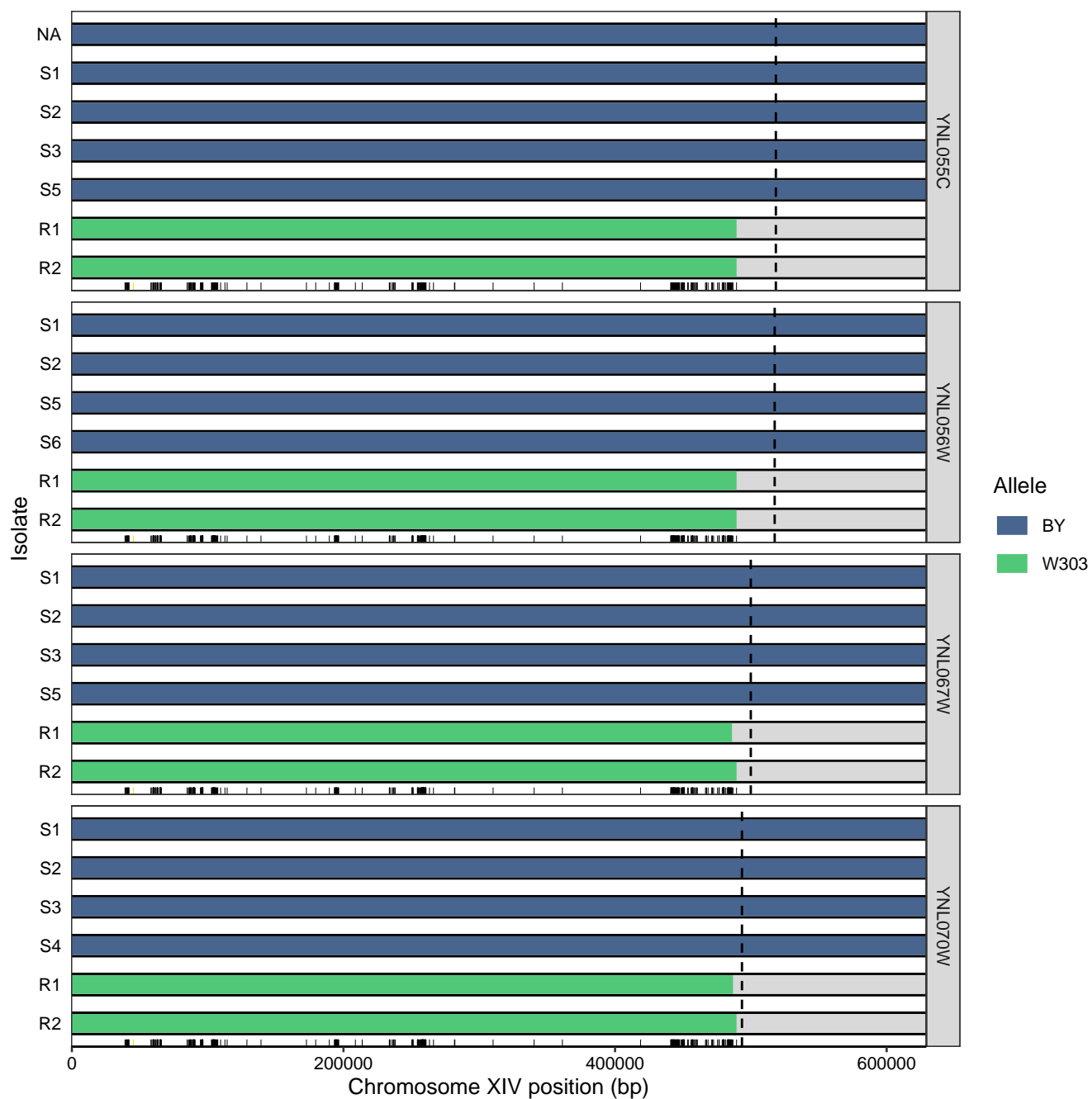

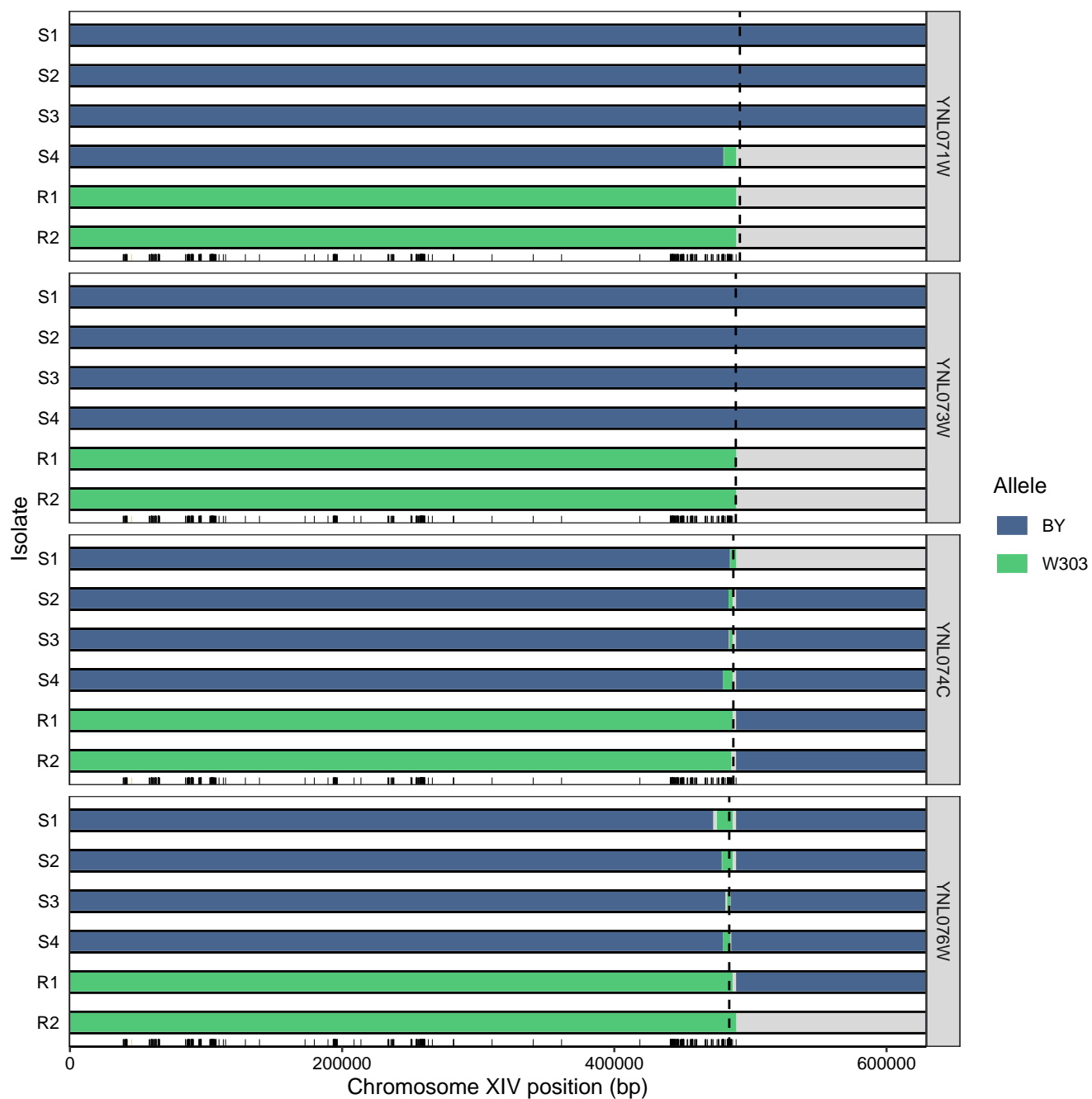

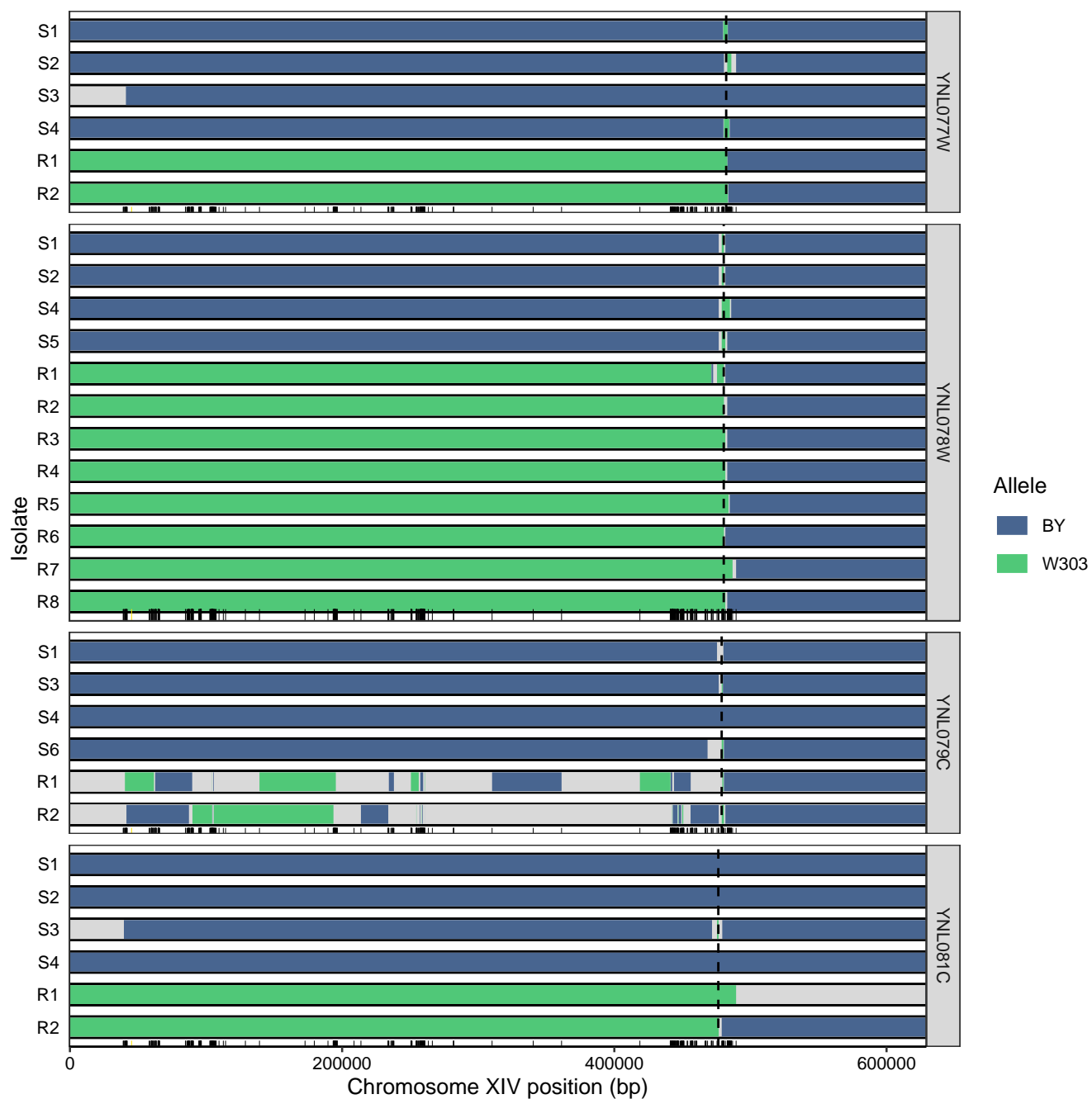

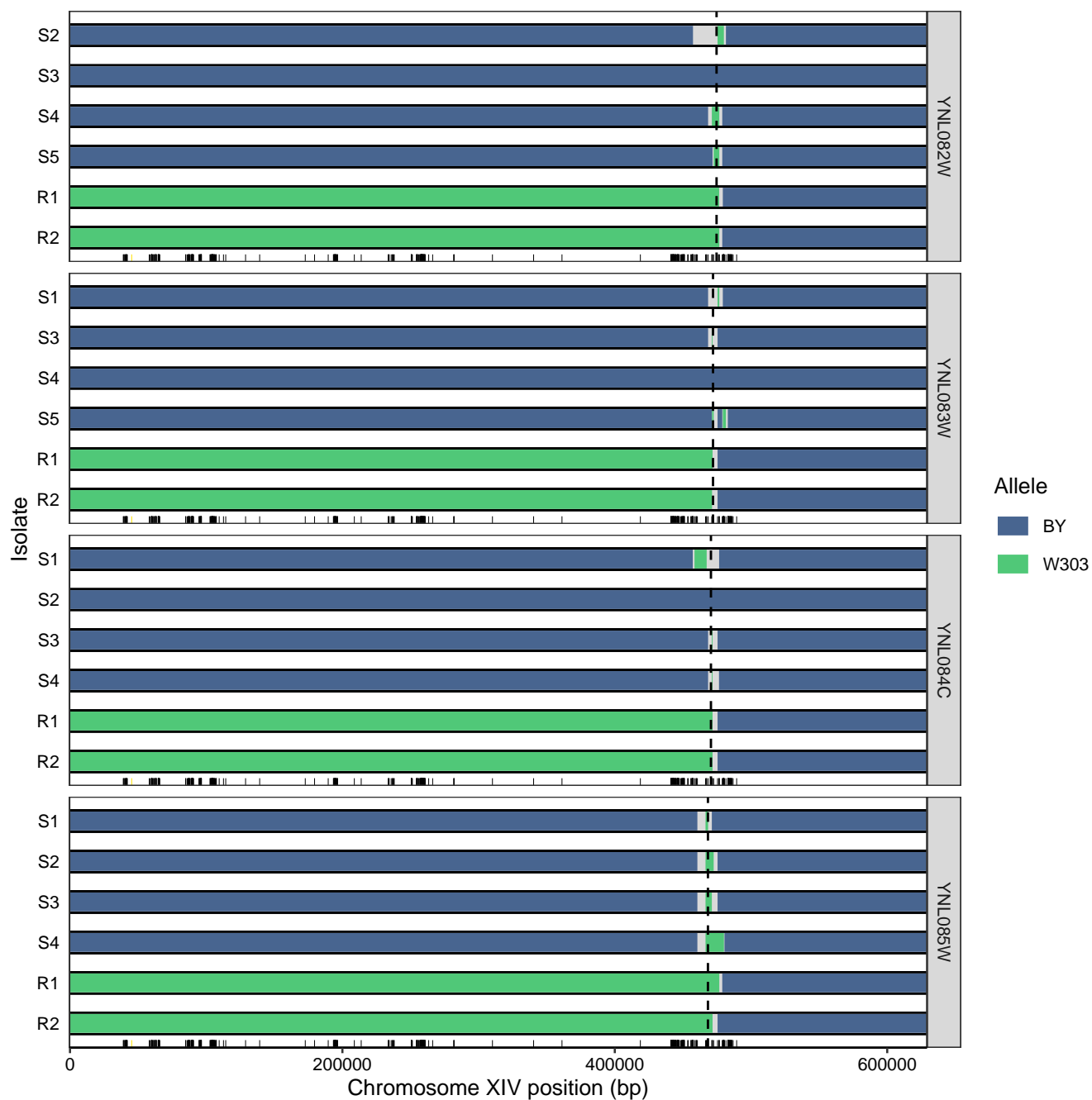

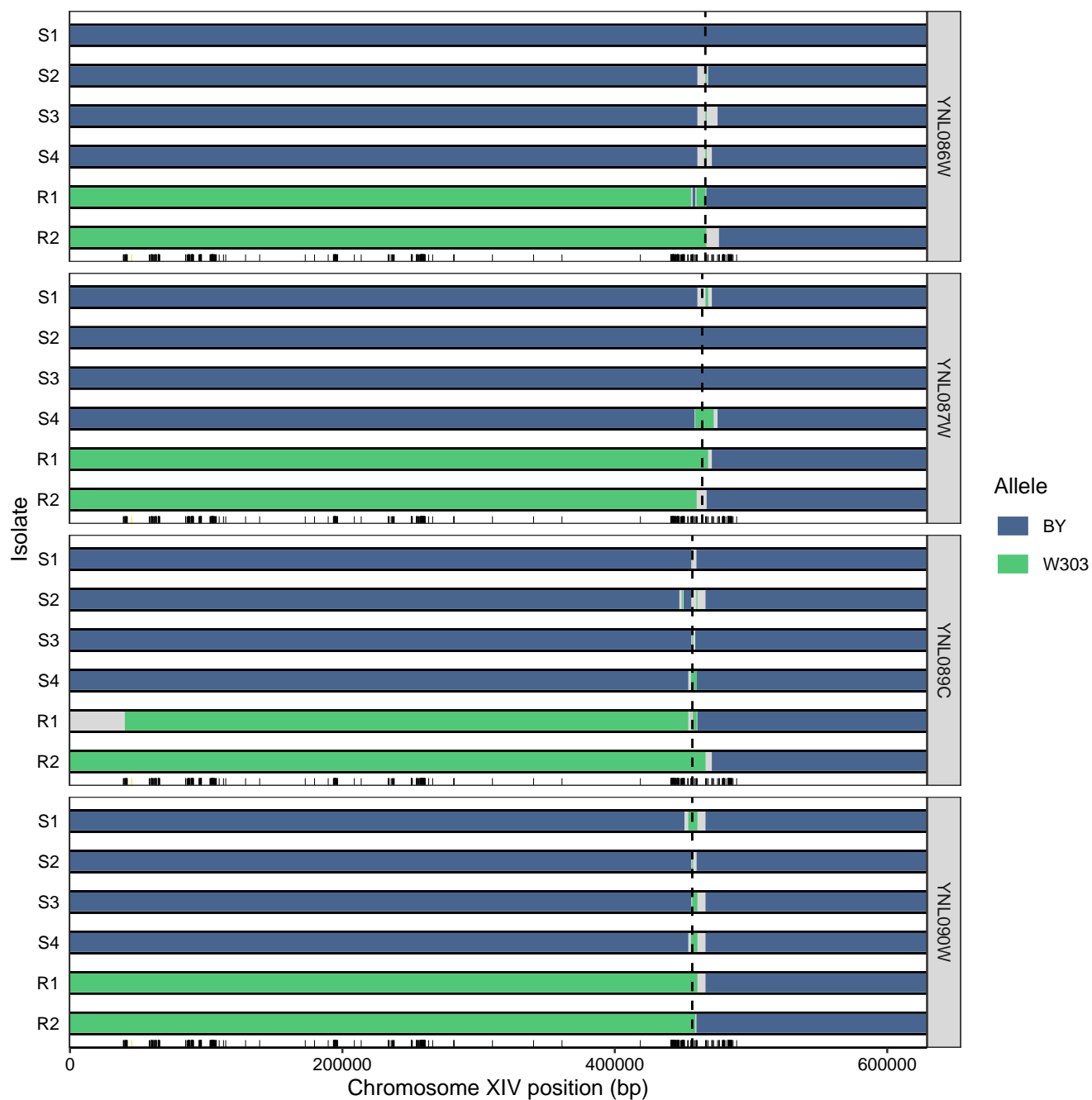

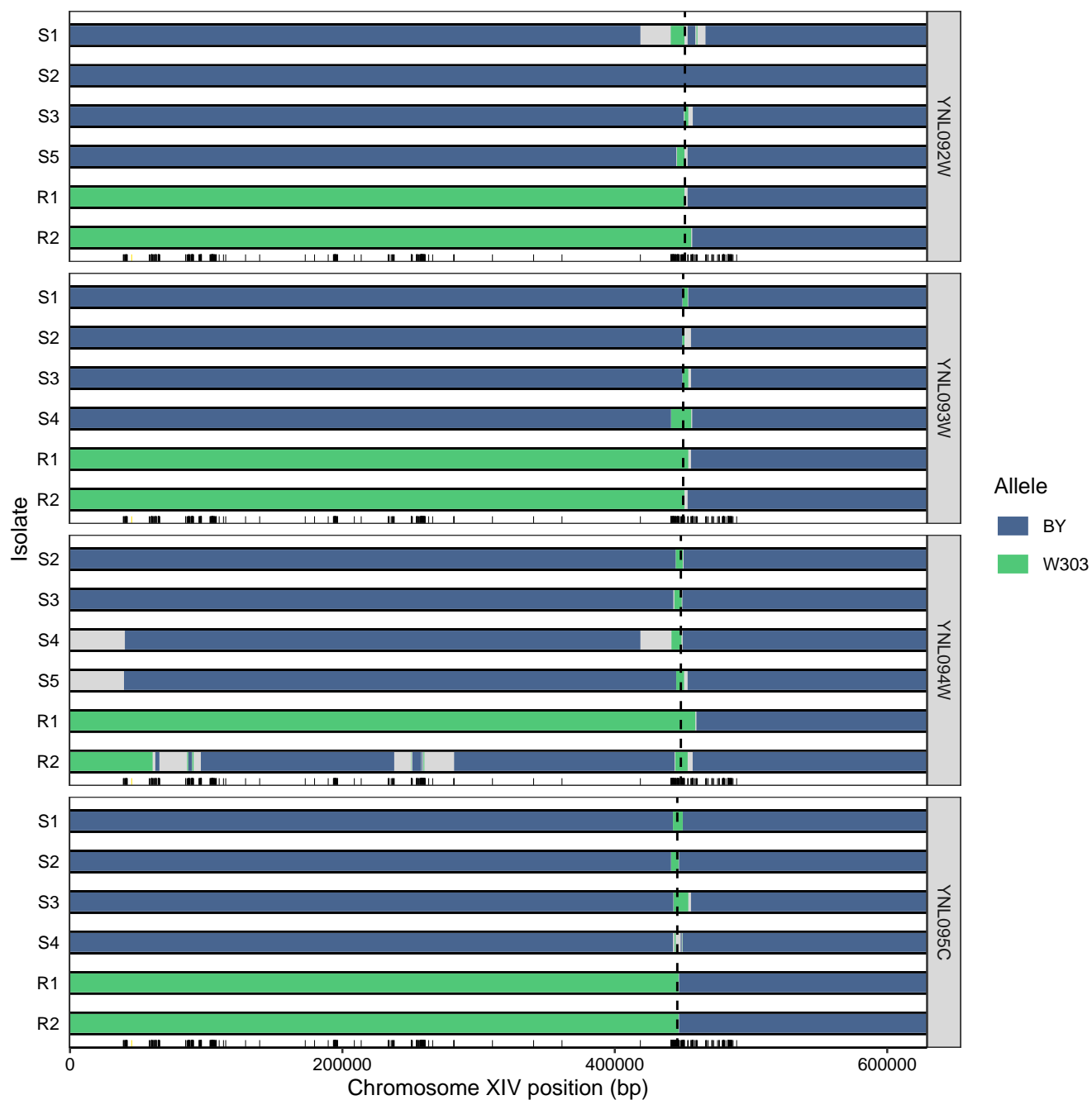

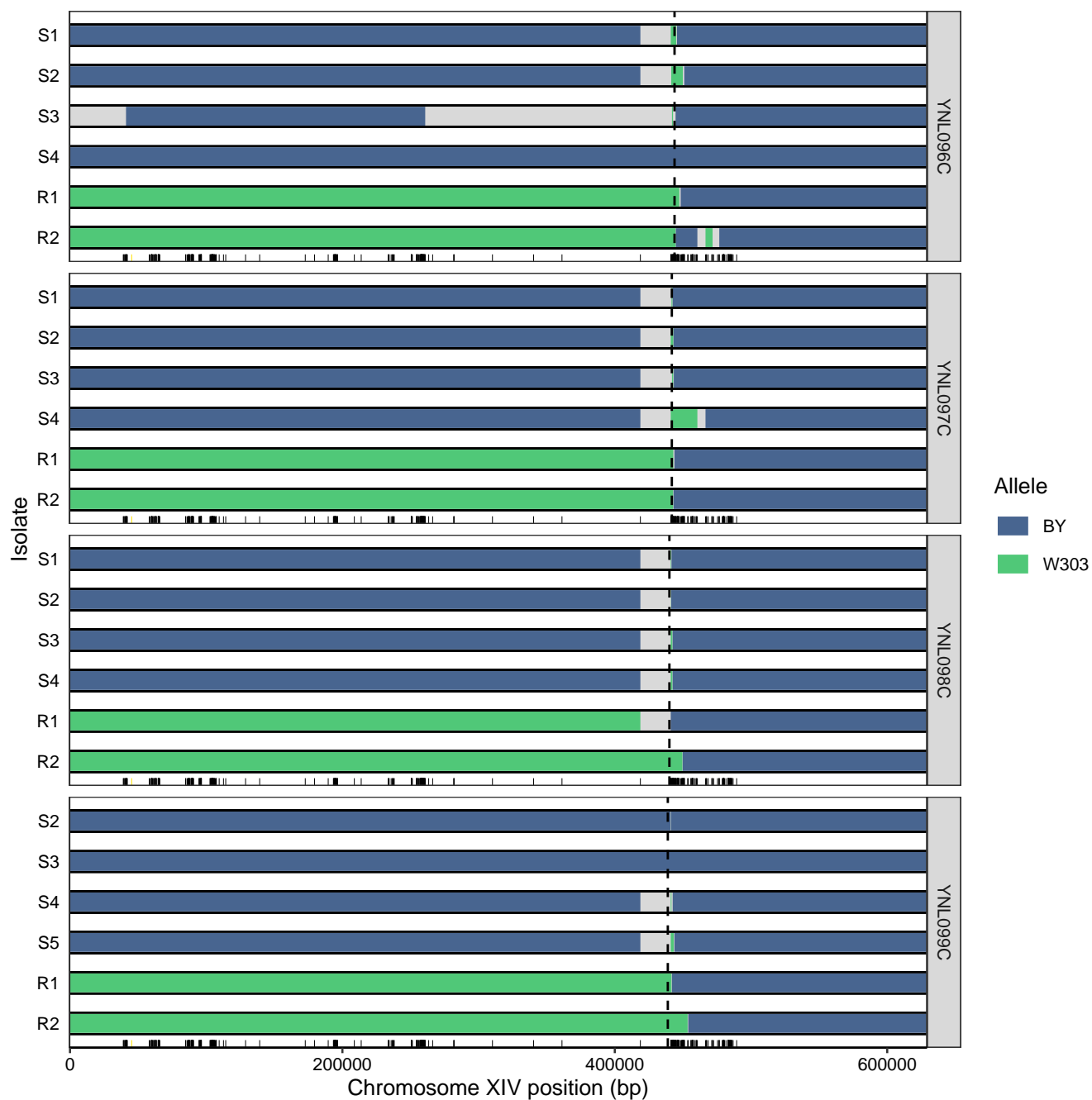

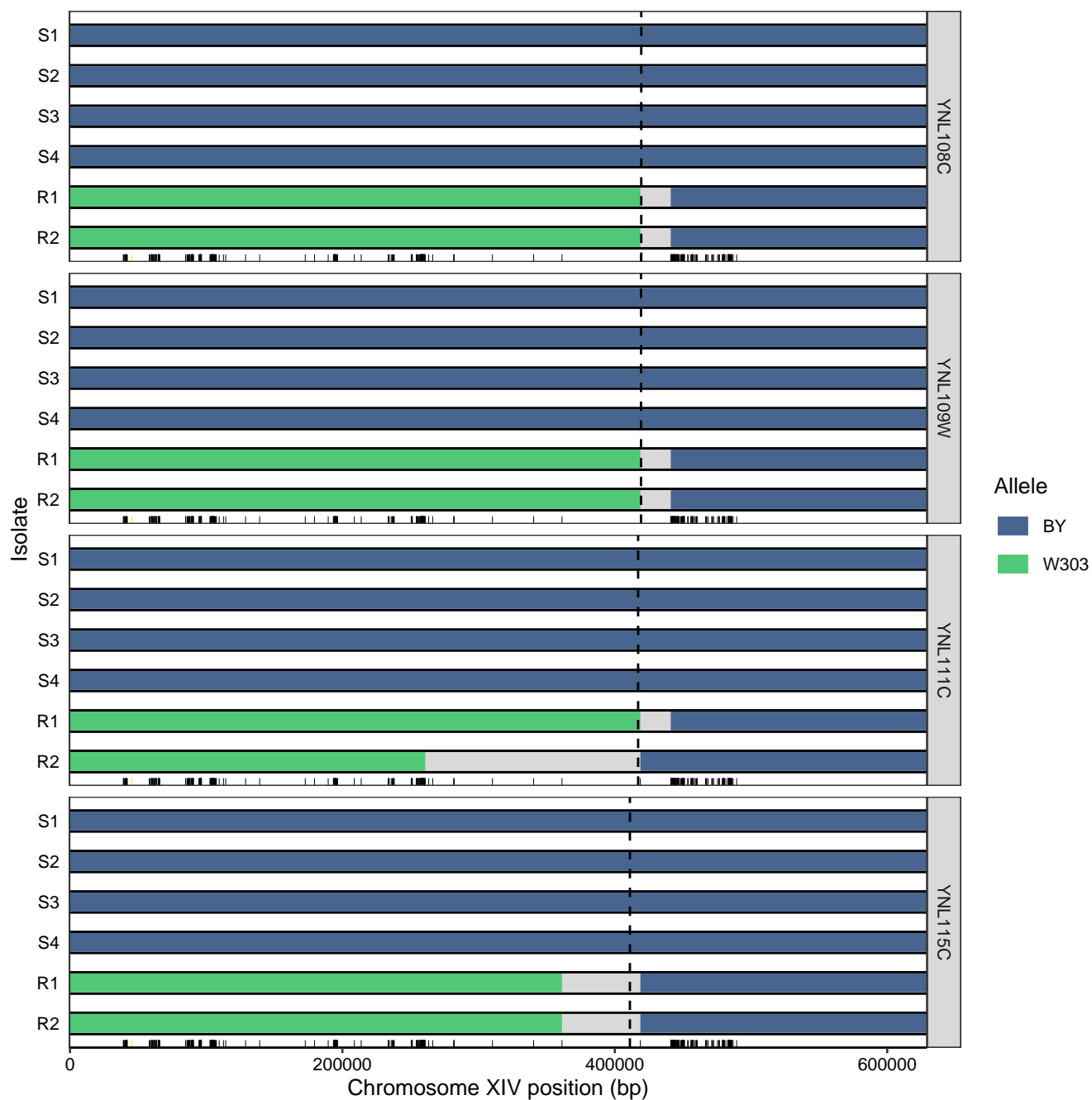

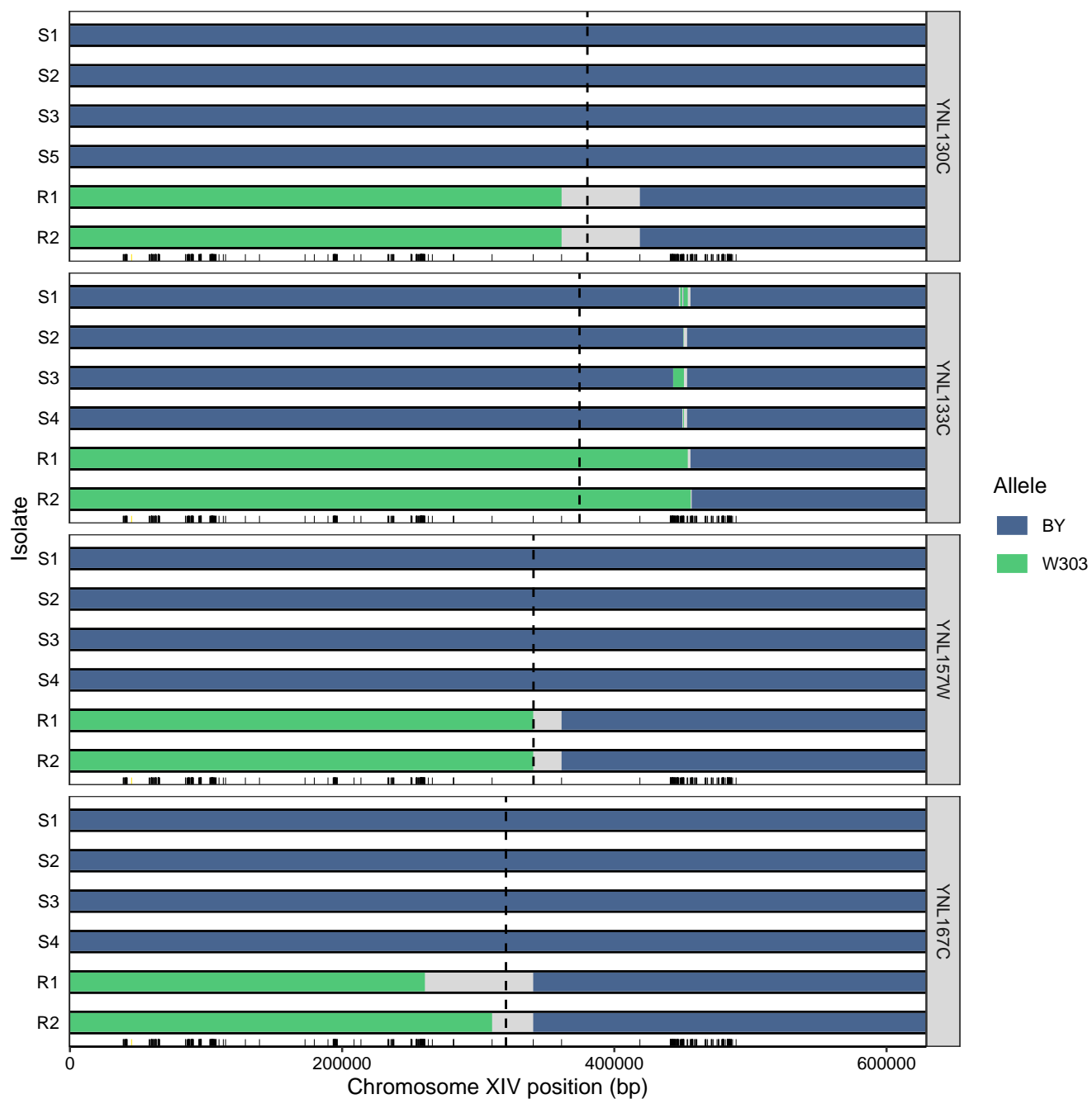

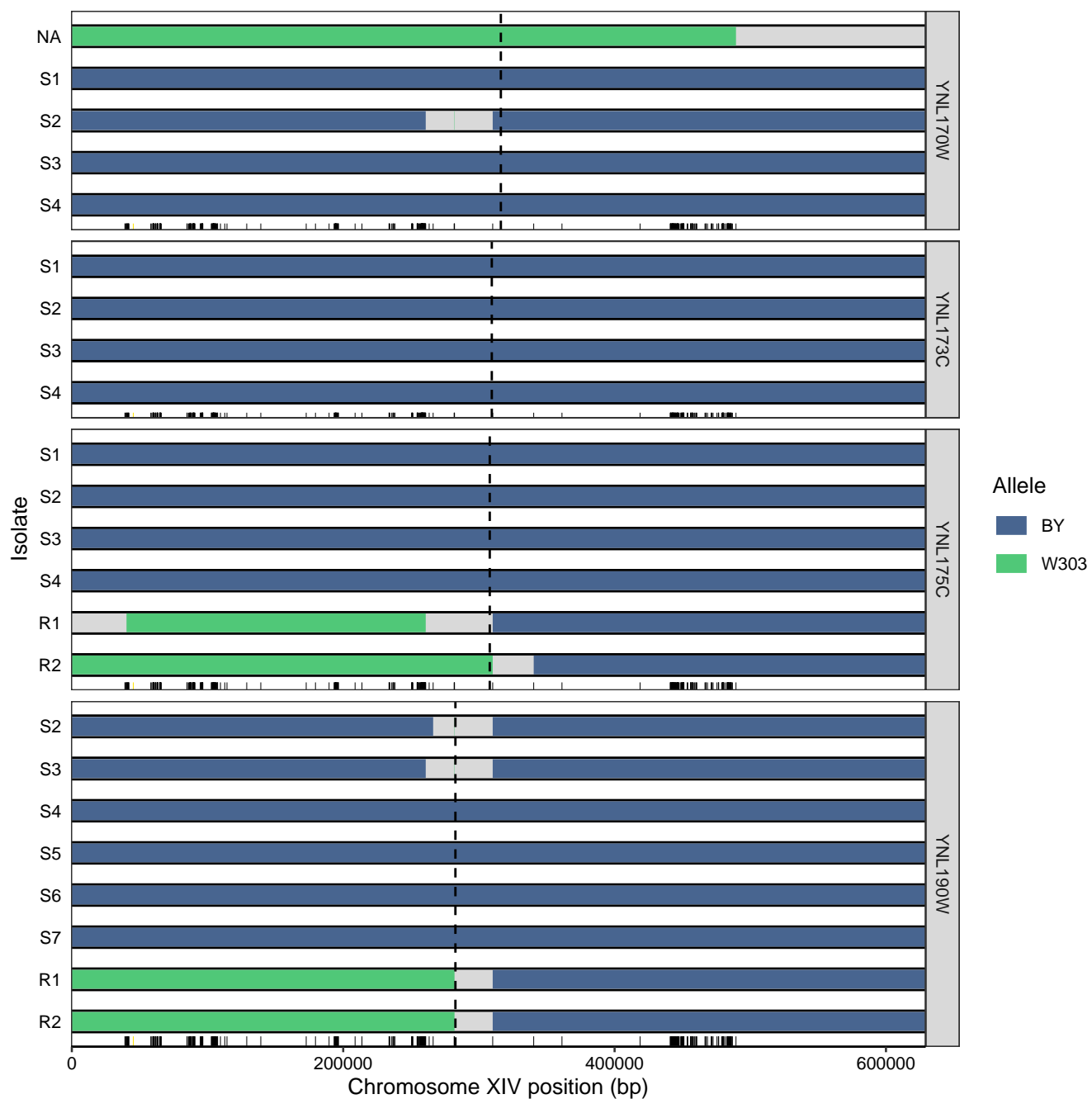

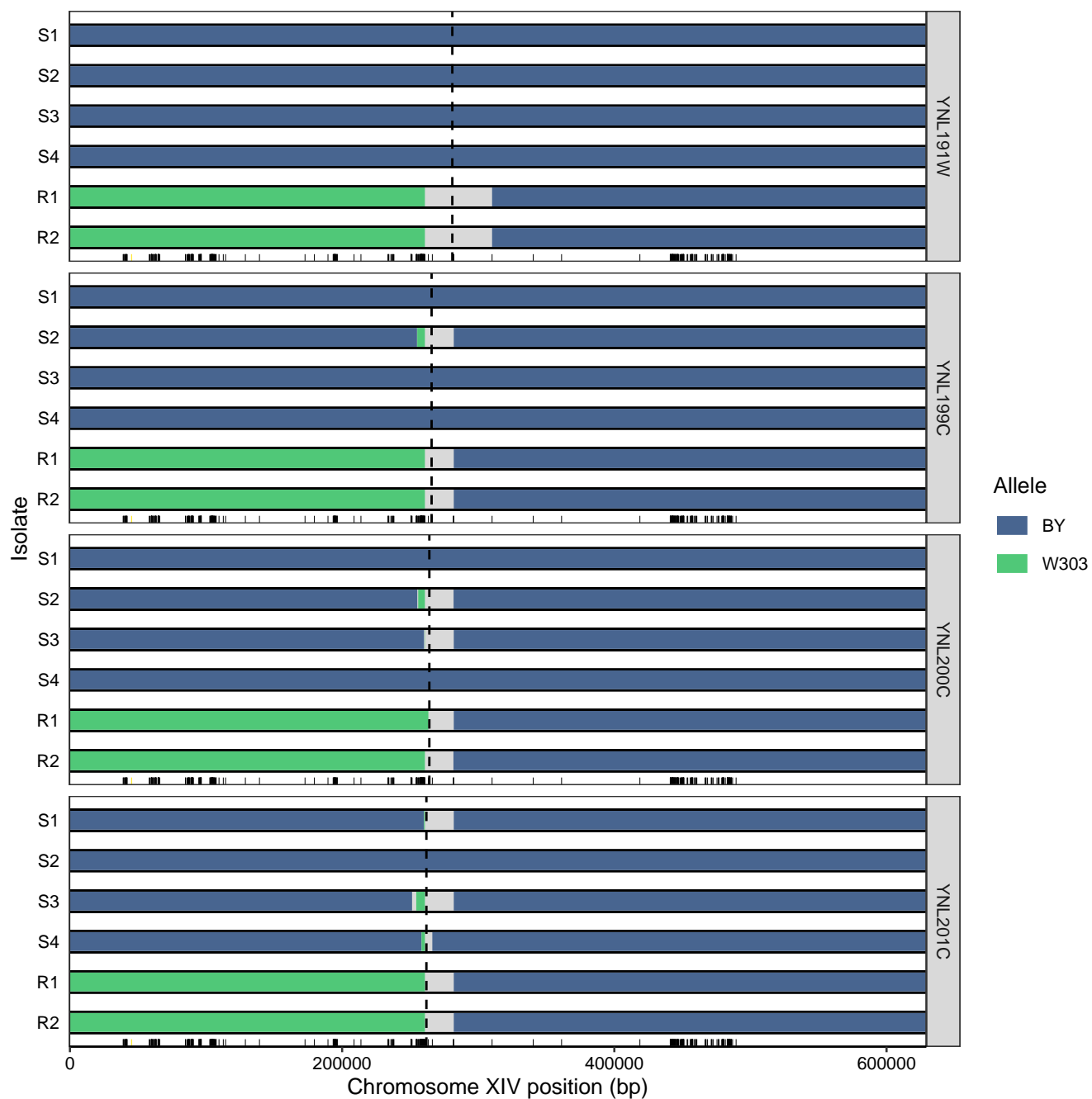

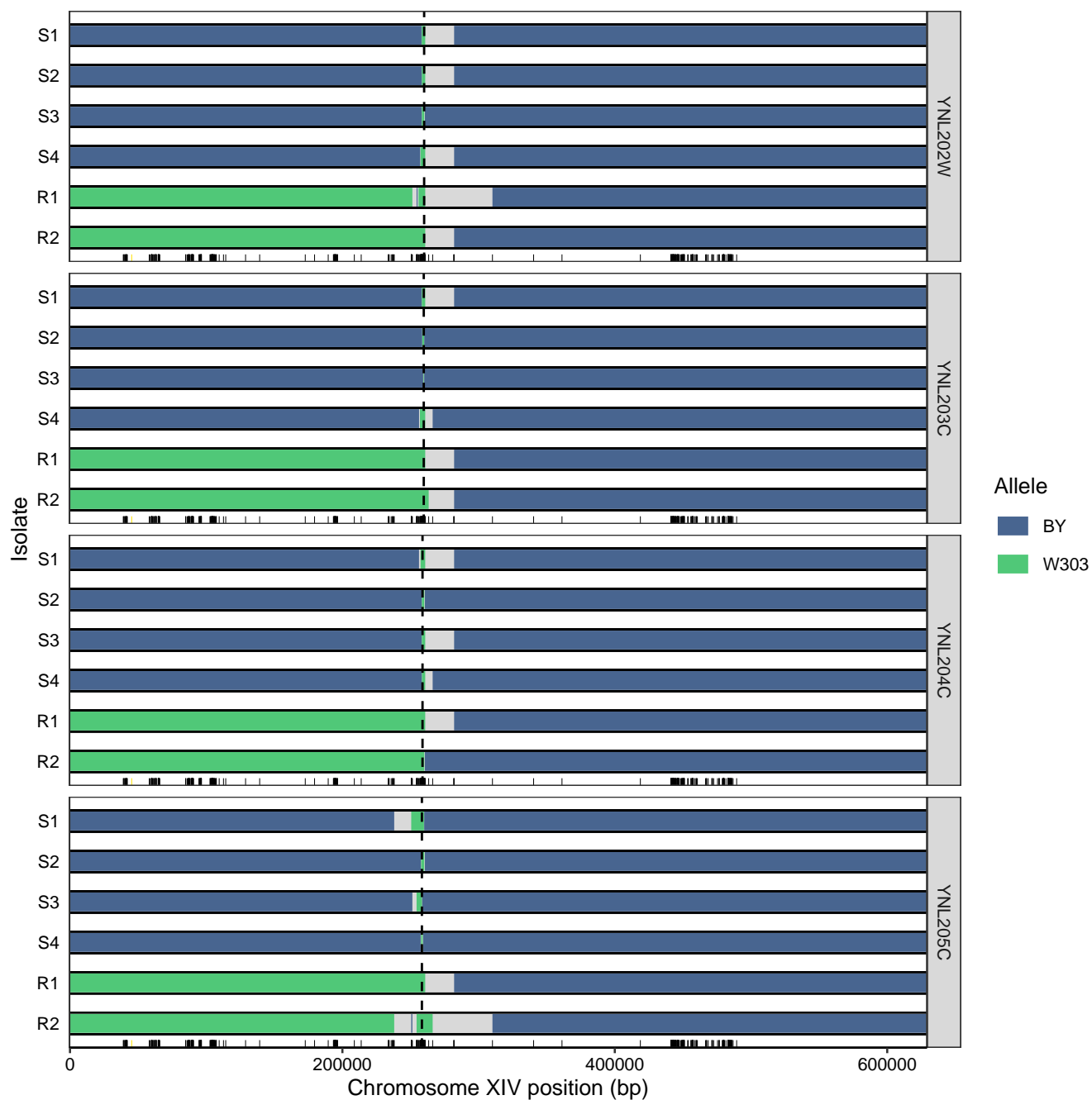

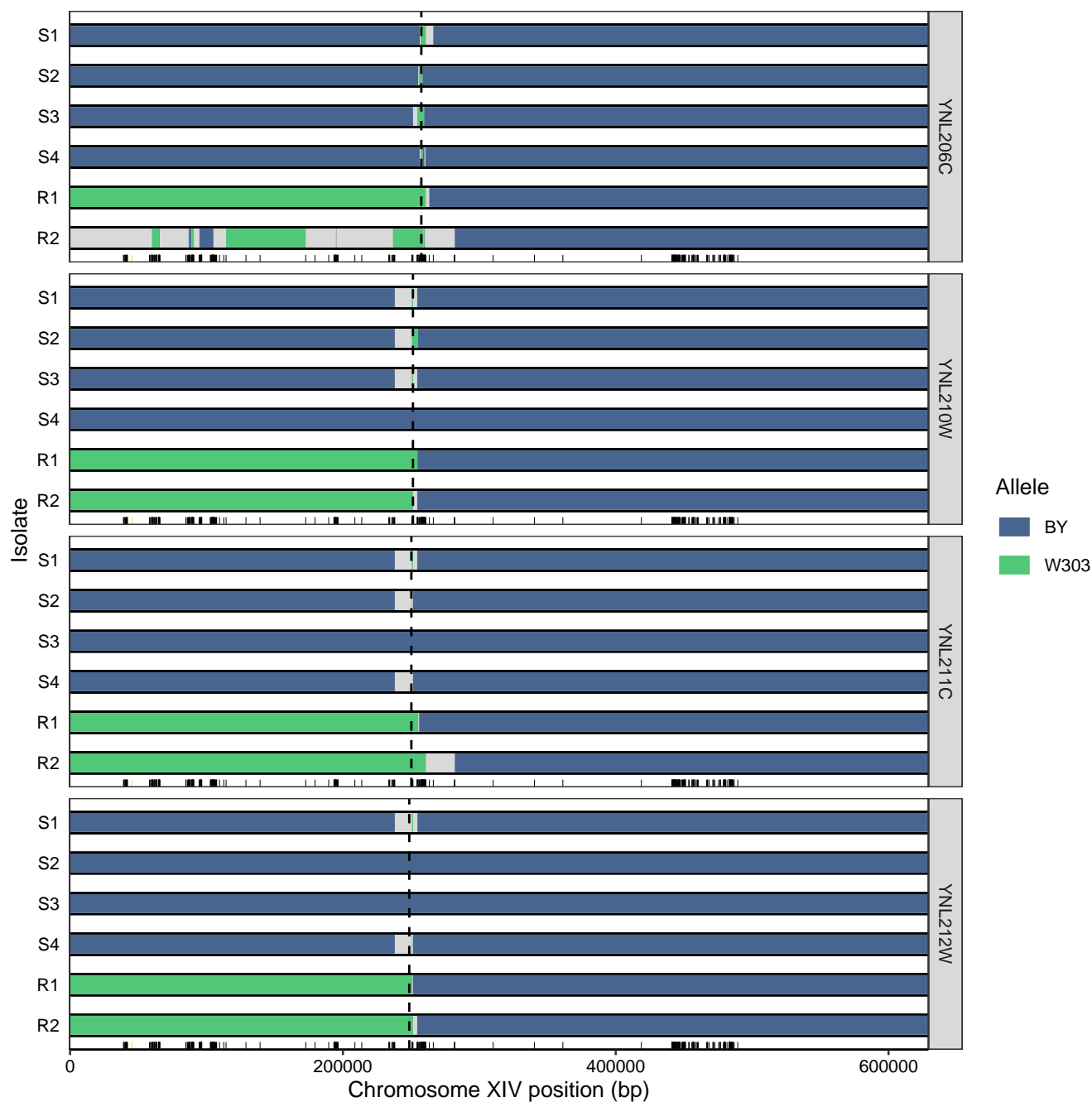

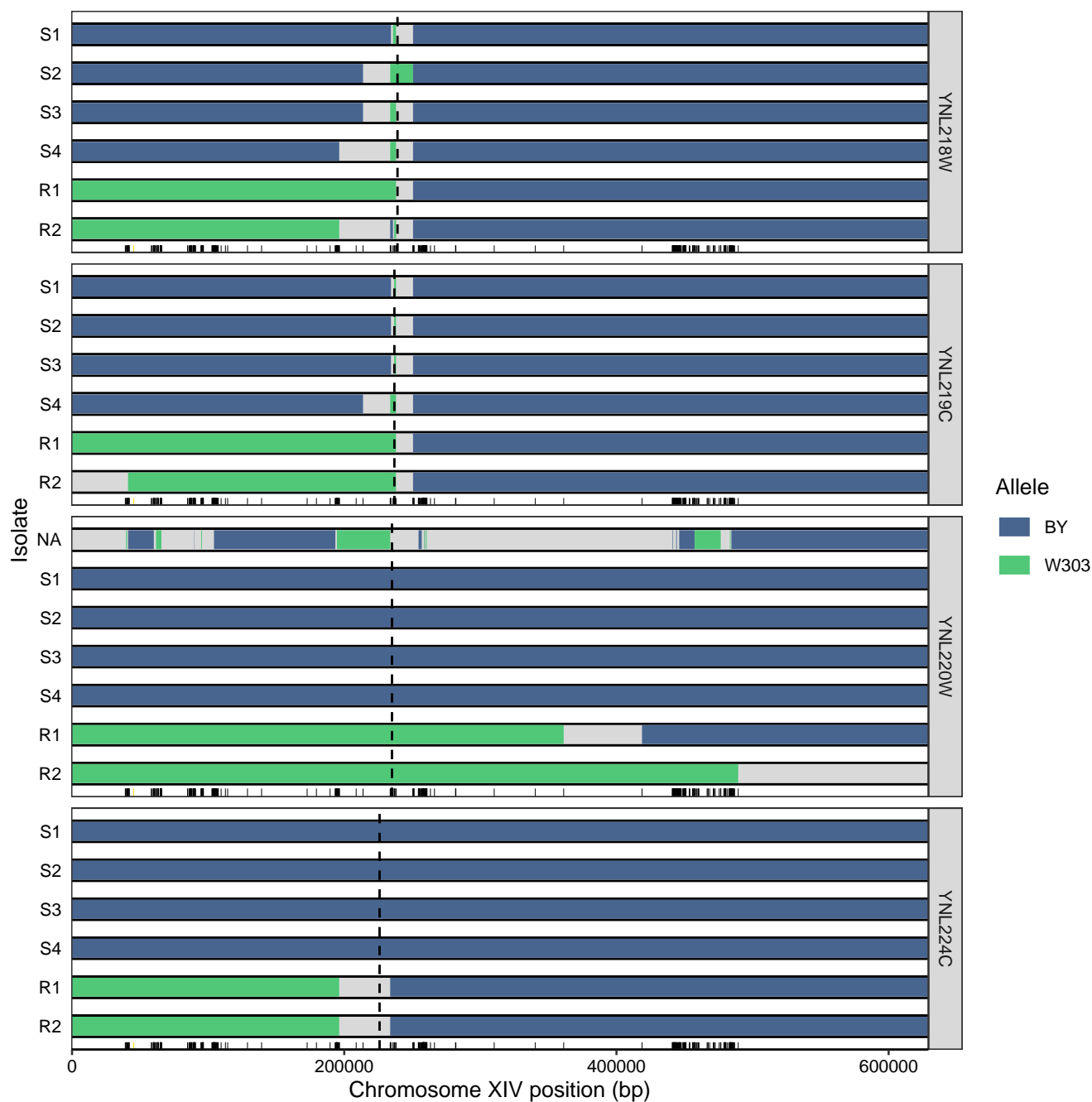

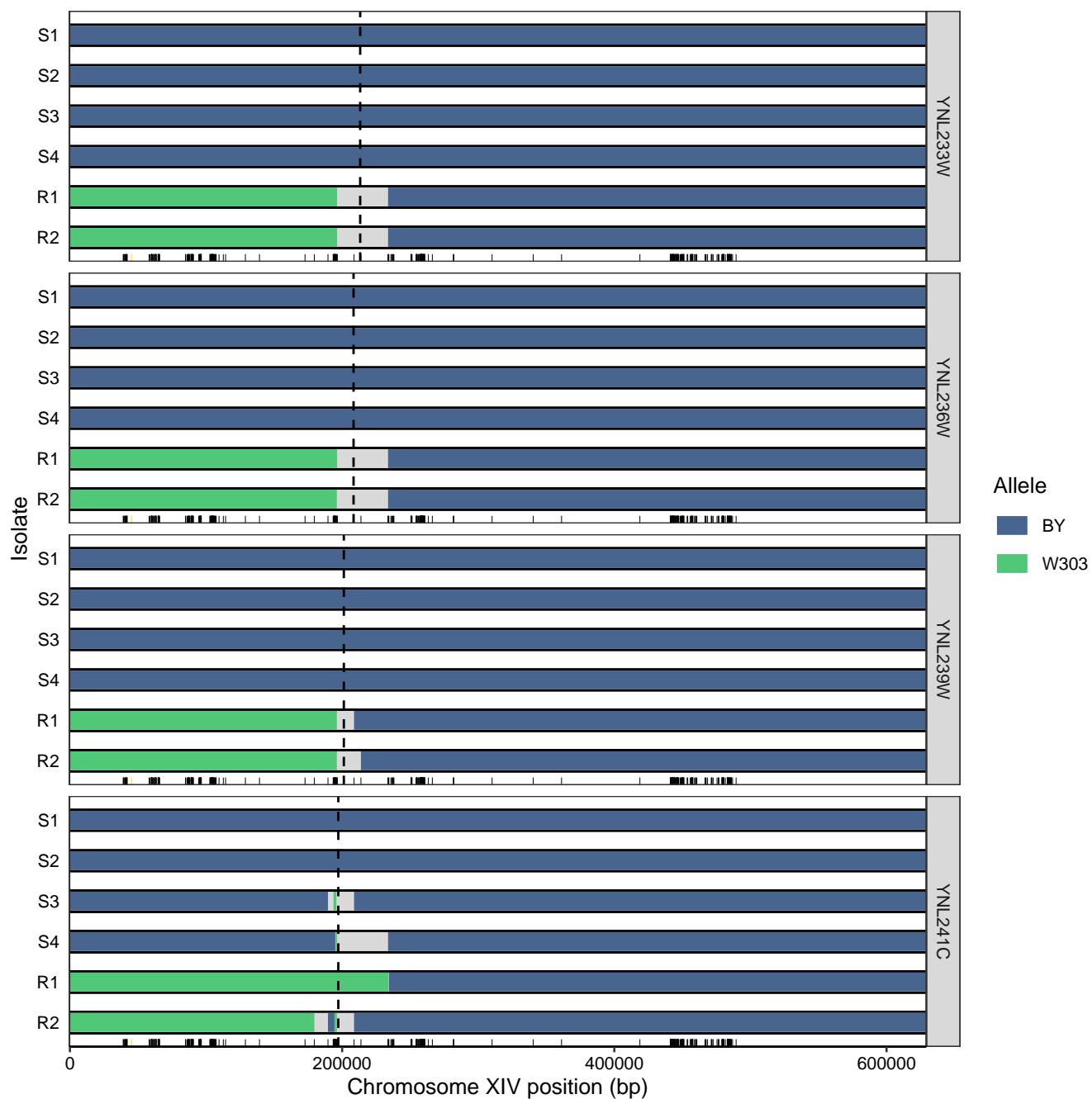

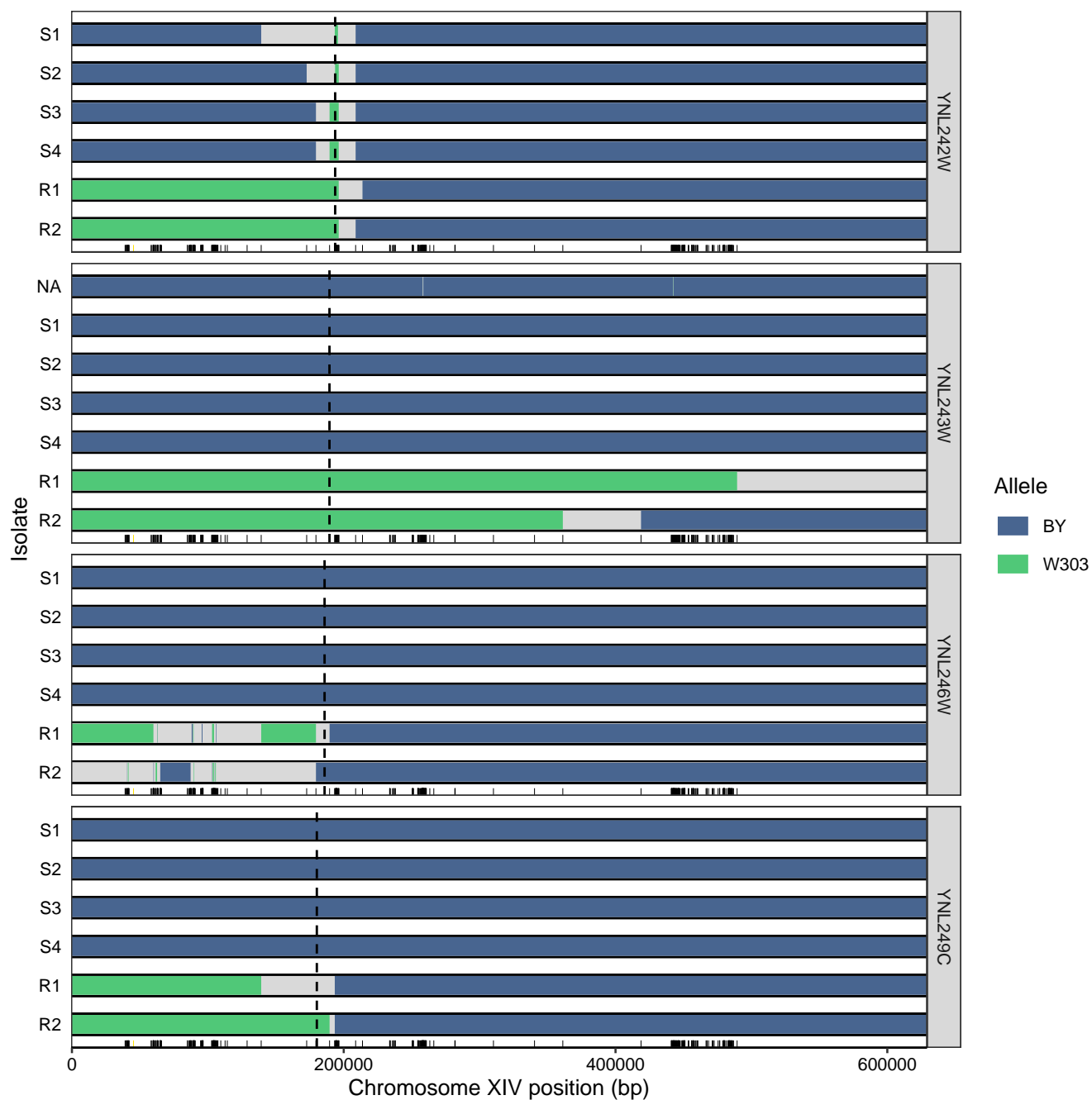

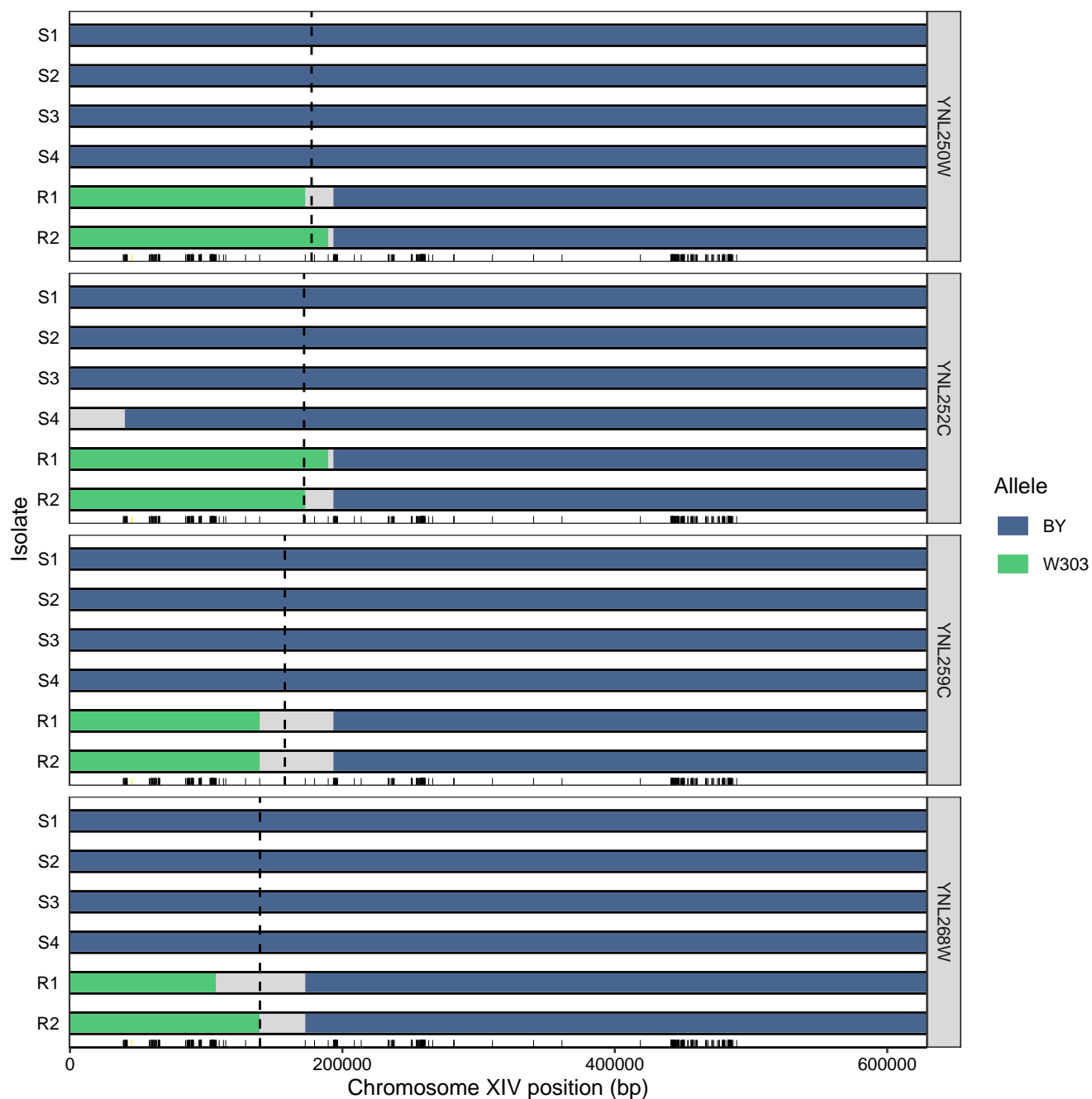

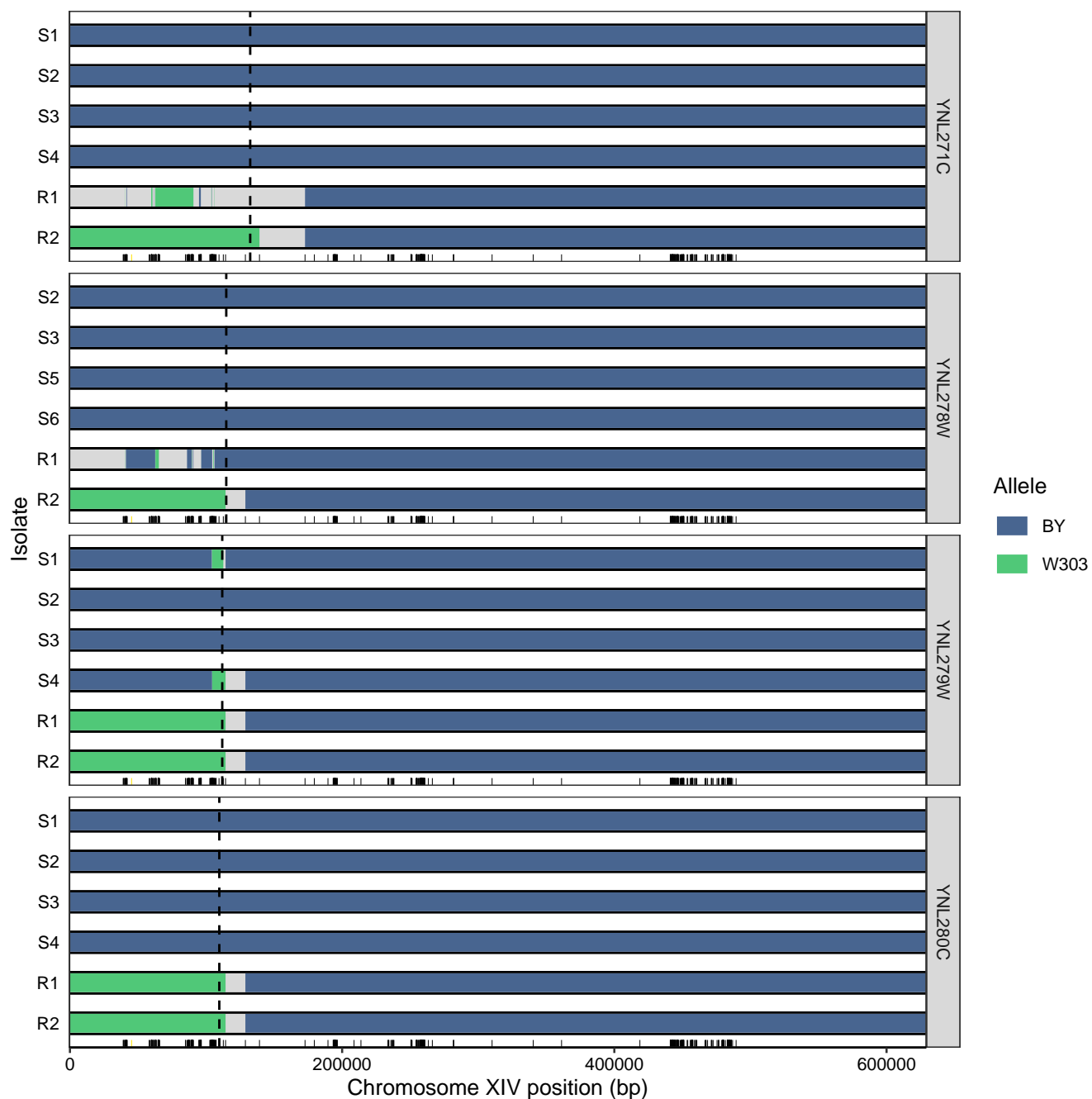

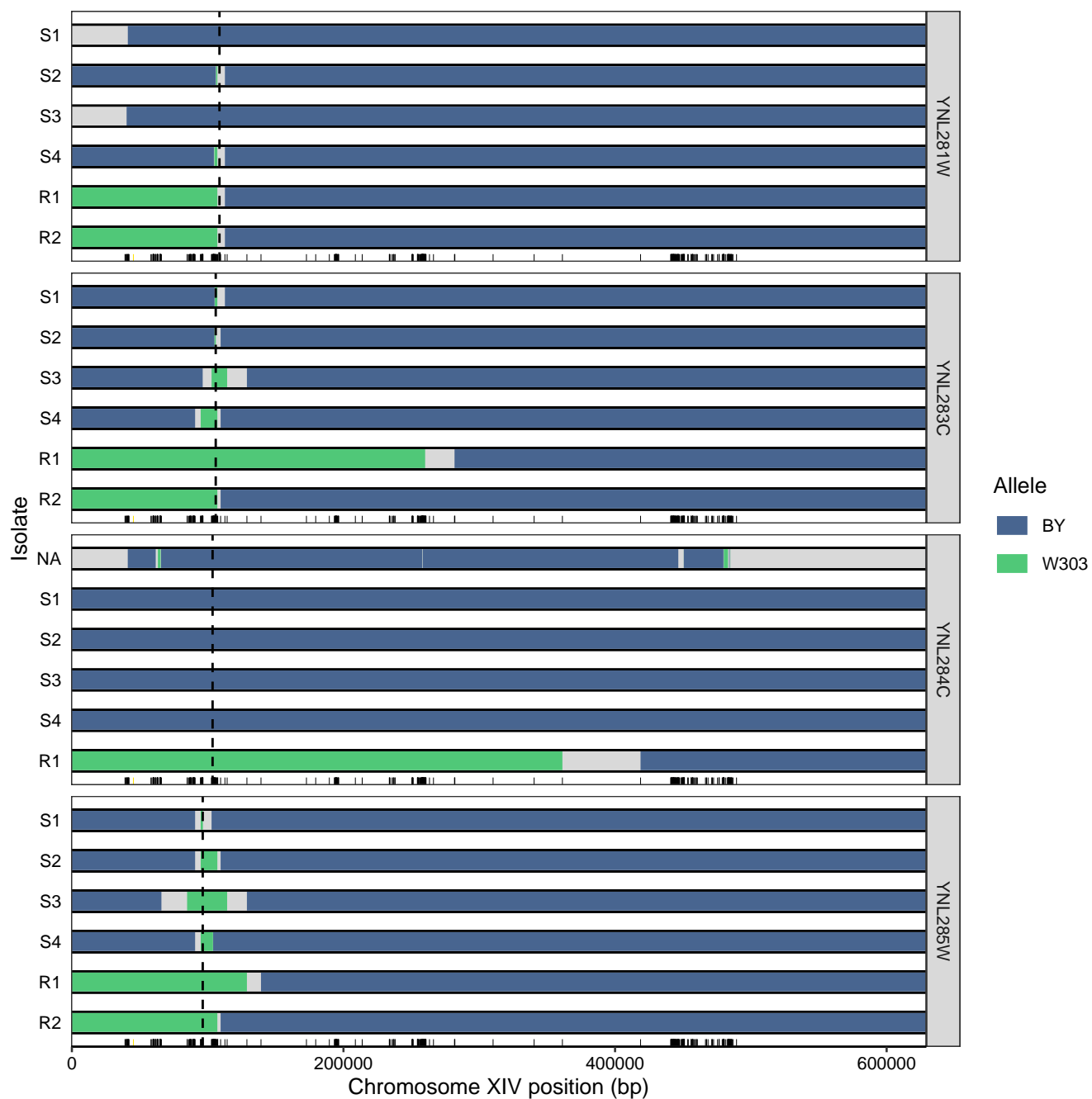

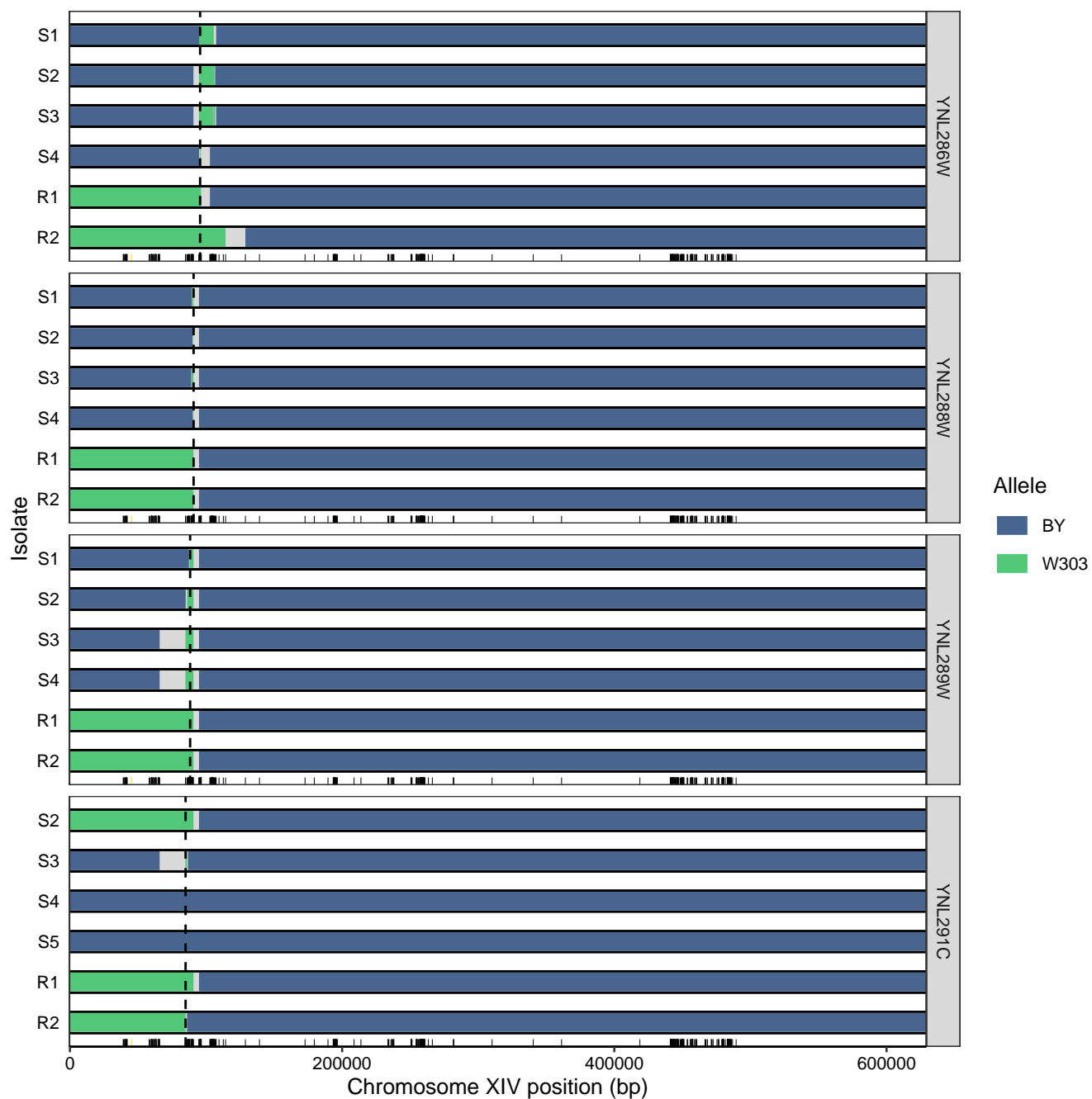

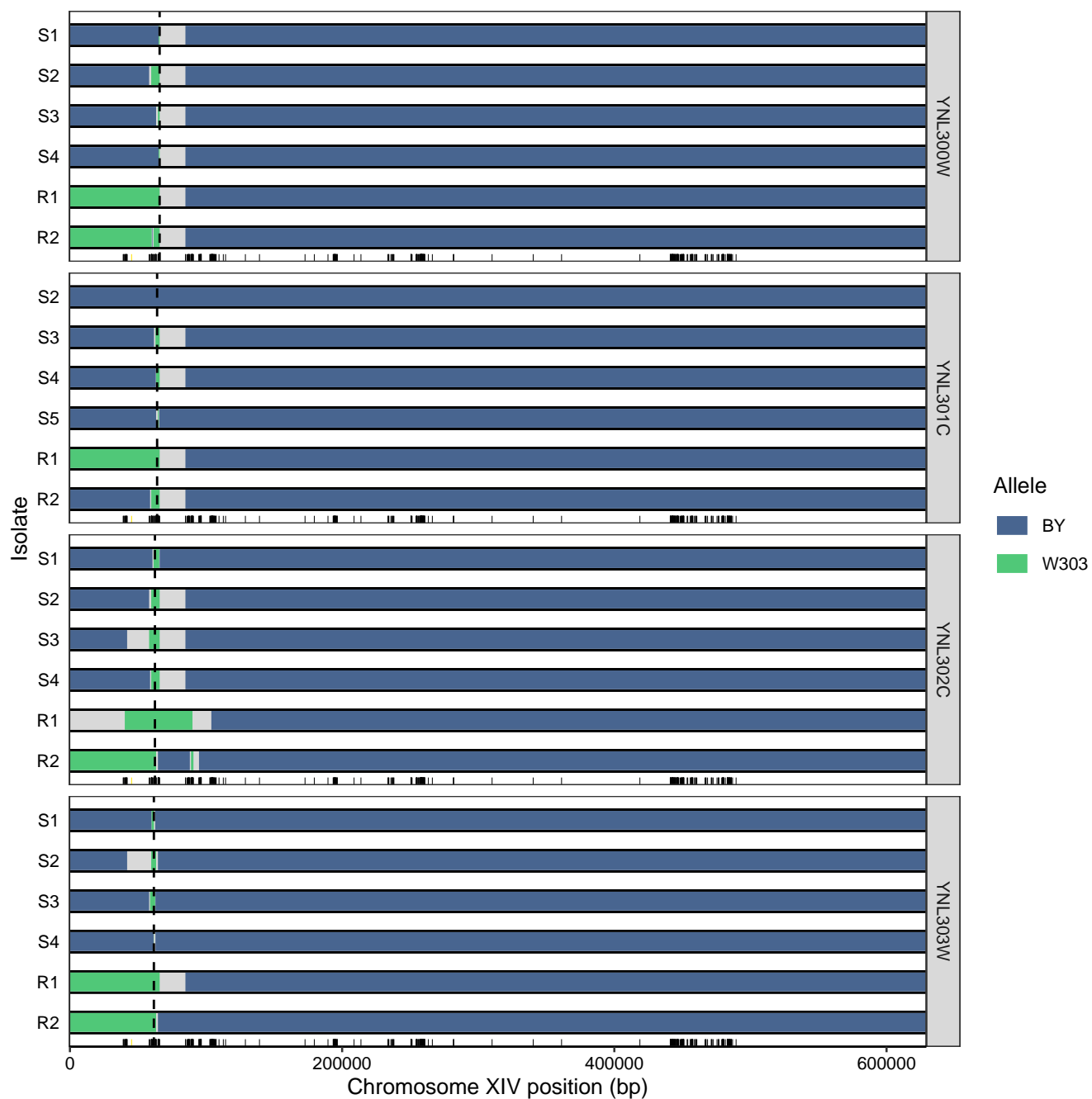

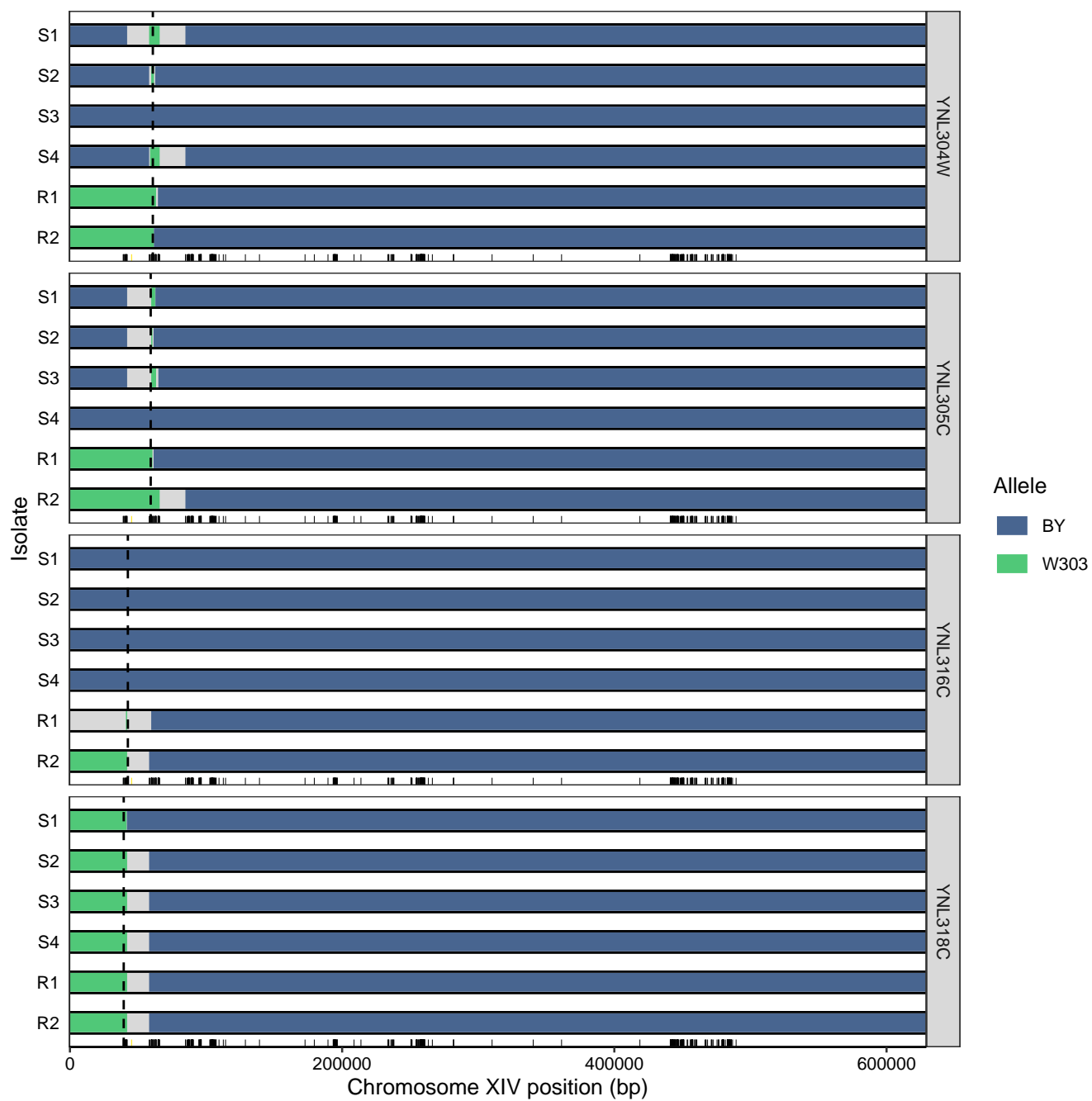

Supplement: Supplement 2 [file media-2.pdf]
